# Novel lipid antigens for NKT cells in cancer

**DOI:** 10.3389/fimmu.2023.1173375

**Published:** 2023-10-16

**Authors:** Michael S. Lee, Tonya J. Webb

**Affiliations:** ^1^ Department of Microbiology and Immunology, University of Maryland School of Medicine, Baltimore, MD, United States; ^2^ Marlene and Stewart Greenebaum Comprehensive Cancer Center, University of Maryland School of Medicine, Baltimore, MD, United States

**Keywords:** NKT cells, cancer immunotherapy, lipid antigens, CD1d, cancer immunosurveillance

## Abstract

Cancer immunotherapy aims to unleash the power of the immune system against tumors without the side effects of traditional chemotherapy. Immunotherapeutic methods vary widely, but all follow the same basic principle: overcome the barriers utilized by cancers to avoid immune destruction. These approaches often revolve around classical T cells, such as with CAR T cells and neoantigen vaccines; however, the utility of the innate-like iNKT cell in cancer immunotherapy has gained significant recognition. iNKT cells parallel classic T cell recognition of peptide antigens presented on MHC through their recognition of lipid antigens presented on the MHC I-like molecule CD1d. Altered metabolism and a lipogenic phenotype are essential properties of tumor cells, representing a unique feature that may be exploited by iNKT cells. In this review, we will cover properties of iNKT cells, CD1d, and lipid antigen presentation. Next, we will discuss the cancer lipidome and how it may be exploited by iNKT cells through a window of opportunity. Finally, we will review, in detail, novel lipid antigens for iNKT cells in cancer.

## Introduction

In 1987, three independent studies were published demonstrating a unique population of mature CD4-/CD8- αβ+ T cells that express a single V_β_ segment and secrete large amount of the cytokine IL-2 following chemical stimulation with PMA and ionomycin (1–3). Subsequent studies found that these CD4-/CD8- αβ+ T cells secreted large amounts of other cytokines, including IFN-γ and IL-4, as well as expressed the cell surface marker NK1.1 (previously only thought to be expressed on NK cells) and showed predominant usage of the V_β_8.2 segment (4–6). In addition, these cells were absent in β_2_-microglobulin deficient mice, suggesting they were MHC class I restricted despite a lack of CD8 expression, being only CD4+ or CD4-/CD8-, which contradicted what was known about T cell restriction at the time (CD8+ T cells are MHC class I-restricted and CD4+ T cells are MHC class II-restricted) (7–9). While the early studies were all conducted in mice, it was also found that a homologous population of CD4-/CD8- and CD4+/CD8- αβ+ T cells that express NK cell markers and are restricted to utilizing the V_α_24-J _α_18 with a limited set of V_β_ fragments existed in humans, and the name NKT cell was finally introduced to describe this unique population of T cells (10, 11).

## NKT cell function

**Figure 1:**
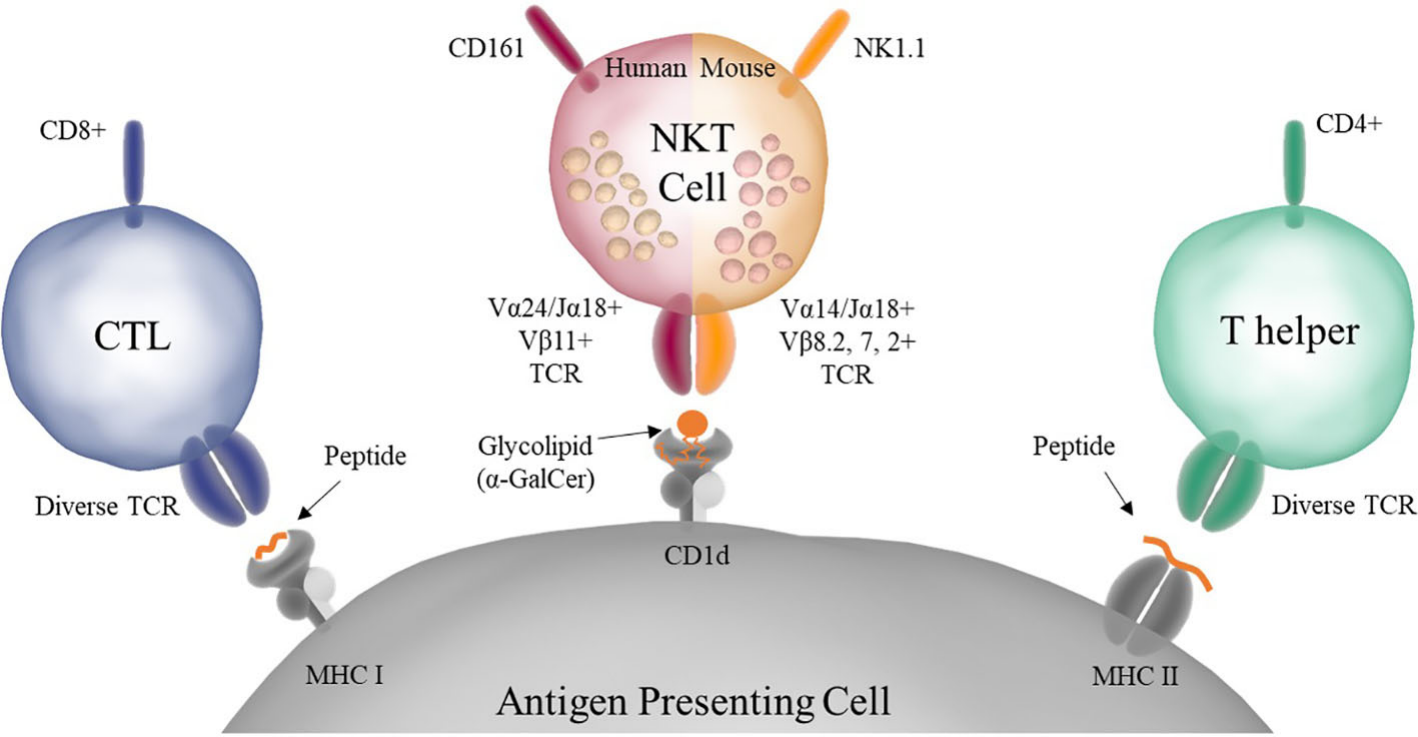
Antigen presentation to conventional T cells and NKT cells. Conventional CD8+, also known as Cytotoxic T Lymphocytes (CTLs), and CD4+, also known as Helper T Cells, have diverse TCRs and recognize peptide antigens presented on MHC I or MHC II, respectively. NKT cells have a semi-invariant TCR that recognizes glycolipid antigens presented on CD1d. Murine NKT cells also express NK markers such as NK1.1. In addition, NKT cells store premade cytokine in intracellular granules that allow them to respond rapidly upon stimulation.

It is now known that NKT cells, despite having a TCR, do not react with peptide loaded onto MHC, but instead react to lipid antigens presented on the MHCI-like molecule CD1d (**Figure 1**). Unlike MHC molecules which present peptides bound into a groove on their surface, CD1d has two deep pockets in which the acyl chains of lipid antigens can bind leaving the head group presented to the NKT cell TCR. In addition, NKT cells are innate-like T cells, as they possess properties that bridge the distinction between the innate and adaptive immune systems (12). NKT cells can be stimulated both through their TCR, via lipid antigens presented on CD1d, and through cytokines (IL-12 and IL-18) secreted by pattern recognition receptor (PRR) stimulated innate immune cells (13, 14). Finally, unlike conventional T cells that require an initial priming step, NKT cells are able to secrete large amounts of cytokine immediately upon stimulation (15).

NKT cells can be divided into subtypes, and further characterized into distinct subpopulations. NKT cells are first split into either type I or type II NKT cells. Type I NKT cells, also referred to as invariant NKT (iNKT), have a semi-invariant TCR consisting of V_α_14-J_α_18 and V_β_ chains of limited diversity, are CD1d-restricted, and respond to the marine sponge-derived glycolipid α-galactosylceramide (α-GalCer) when bound to CD1d. Type II NKT cells are also CD1d-restricted but have diverse TCRs and do not react to α-GalCer, but instead react with other self-lipids such as sulfatide. Our lab recently published an extensive review on NKT cell subsets and their development (16). This review will focus on Type I iNKT cells.

**Figure 2:**
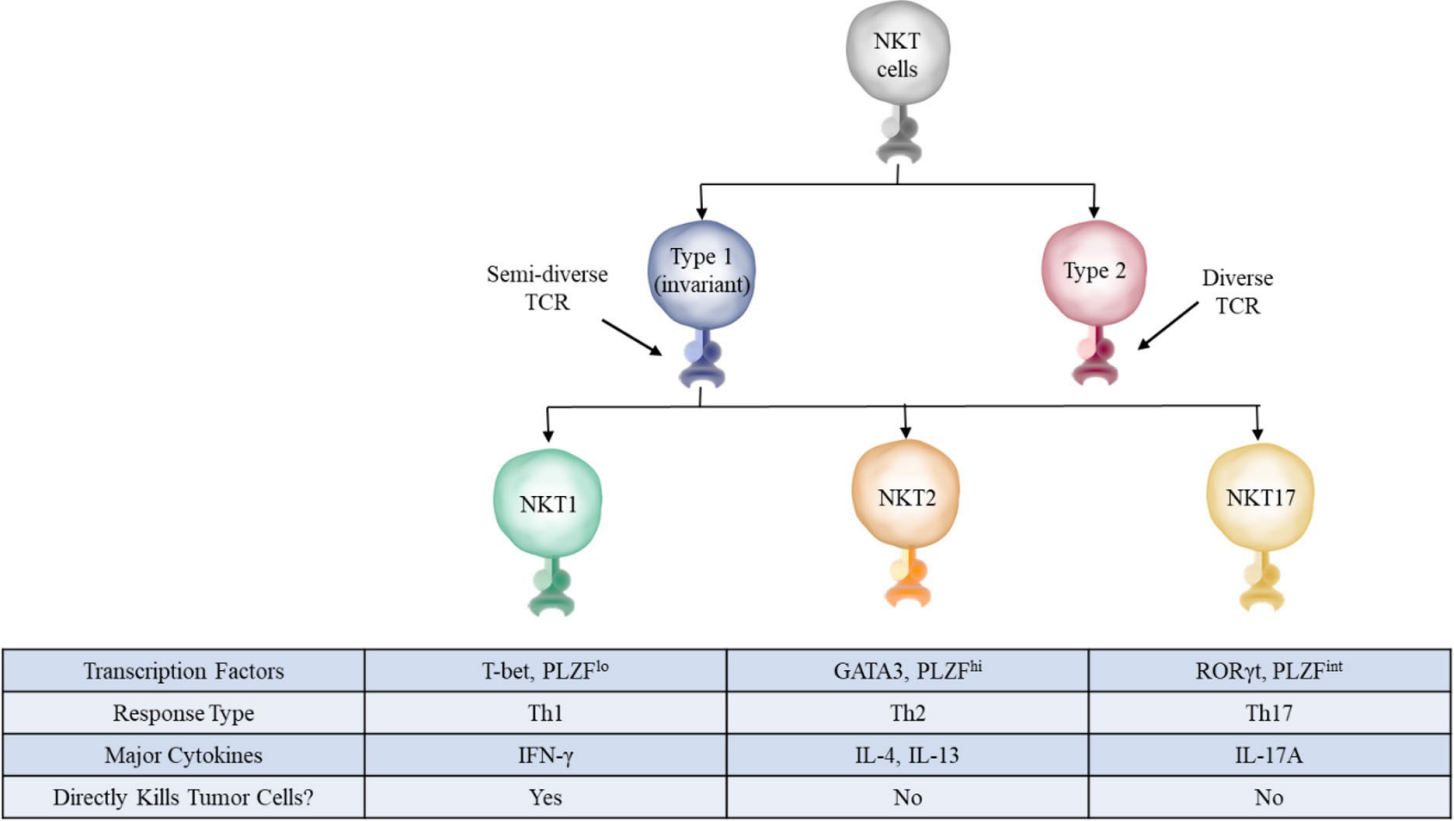
NKT cell subsets. The three main subsets of NKT cells in mice have distinct defining transcription factors, surface markers, and cytokines. These subsets closely mirror those found in conventional T cells, with NKT1, NKT2, and NKT17 roughly corresponding to Th1, Th2, and Th17 cells, respectively.

Type I iNKT cells can be further subdivided into multiple categories: NKT1, NKT2, and NKT17 (**Figure 2**) (16, 17). These categories reflect the type of cytokines they secrete upon stimulation, with IFN-γ, IL-4, and IL-17 respectively being the classic cytokines secreted by these different cells. These different subtypes follow the traditional framework of Th1, Th2, or Th17 type responses found in conventional helper T cells and innate-lymphoid cells (ILCs). The Th1 subtype is generally considered to be optimized against intracellular threats such as viruses in addition to tumor cells, while the Th2 subtype targets extracellular parasites, and the Th17 subset specializes against extracellular bacteria and fungi (18, 19). However, unlike conventional T cells which adopt Th1, Th2, or Th17 properties depending on the cytokines present during their initial stimulation, NKT cells adopt these properties during their development, further cementing their nature as an innate-like T cell (16). With respect to function in anti-tumor immunity, type I and type II NKT cells often have opposing roles, with type I NKT cells generating a pro-inflammatory anti-tumor response and type II NKT cells generating an anti-inflammatory pro-tumor response (15).

## NKT cells and the anti-tumor response

A 2002 study demonstrated direct evidence that NKT cells can participate in the anti-tumor response (20). The study utilized the C57BL/6 TCRJα281-/- knockout mouse, which is deficient in NKT cells due to the absence of Jα281 (now known as Jα18), the gene encoding the Jα portion of the *i*NKT TCR. Wild type C57BL/6 and Jα281-/- mice were subcutaneously implanted with a sarcoma cancer cell line and tumor growth was measured over time. While the tumors did not grow in wild type mice, they grew well in NKT cell deficient mice. To confirm that NKT cells were responsible for controlling tumor growth, additional studies were conducted in which NKT cell deficient mice received increasing numbers exogenous NKT cells. The authors observed a concomitant decrease in tumor growth as the numbers of adoptively transferred NKT cells increased.

It is now known that, in the context of the anti-tumor immune response, iNKT cells can directly and indirectly target cancers (21). They can directly lyse tumor cells through interactions such as Fas/FasL, TRAIL, and perforin/granzyme (22–24). They can indirectly target tumor cells through the secretion of large amounts of cytokines, such as IFN-γ, that stimulate other arms of the immune system, including macrophages, NK cells, and CTLs, to attack tumor cells (25–27). The unique combination of properties present in iNKT cells makes them promising tools to be used in cancer immunotherapy. Indeed, studies aimed at boosting iNKT cell number and function using α-GalCer-loaded DCs as a cancer immunotherapy in mice and humans have found that higher in iNKT cell numbers correlate with improved outcomes, although translation to improved outcomes have not been as clearly demonstrated in humans (28–33).

The field of immunotherapy has grown exponentially since its inception to include a variety of different approaches. While fields like checkpoint therapy, modified APCs, and CAR-iNKT cells remain as important fields of research, with new targets and more advanced techniques are areas of active research, there are many possible unique mechanisms that tumors can utilize to ultimately evade immune destruction. In fact, based on what we know about the immunoediting process, it is expected that a tumor could eventually acquire the ability to evade any specific immunotherapy. Therefore, it is imperative that we continue to develop new treatments, expanding the toolbox available to physicians allowing them to be prepared for new developments. However, in order to develop novel treatments, we first need to understand how different evasion mechanisms work.

## CD1d-mediated antigen presentation

Like classical class I molecules, the nascent CD1d α-chain in the endoplasmic reticulum (ER) interacts with the chaperon proteins calnexin, calreticulin, and Erp57 help to ensure proper protein folding (34, 35). The CD1d α-chain then associates with β_2_-microglobulin to form a complete CD1d molecule, although some evidence exists suggesting the CD1d α-chain can be expressed on the plasma membrane independent of β_2_microglobulin (36). Microsomal triglyceride transferase (MTP) likely helps to load endogenous lipids onto the newly formed CD1d in order to stabilize the binding pockets (37). CD1d then traffics from the ER through the Golgi network to the plasma membrane (PM).

CD1d then recycles from the PM into endocytic compartments where it exchanges its loaded endogenous lipid for lipids processed in those compartments before returning to the PM (38). This process depends on the presence of a tyrosine based signaling motif as well as the invariant chain (39). The role of invariant chain, traditionally associated with MHC II, in CD1d trafficking is unclear. CD1d co-precipitates with the invariant chain and invariant chain deficient DCs had reduced capacity to stimulate self-reactive NKT cells (39, 40). Additionally, loss of cathepsin S, an enzyme involved in invariant chain processing, resulted in altered CD1d trafficking and defects in CD1d presentation to NKT cells (41). However, invariant chain deficient mice showed no defects in NKT cell numbers or CD1d-mediated antigen presentation to NKT cells by primary splenocytes (42). The latter finding was subsequently challenged when invariant chain deficient mice were shown to have reduced thymic NKT cell percentages, reduced Vβ8+ usage, and defective responses against *Mycobacterium tuberculosis* (43).

After CD1d has been internalized from the plasma membrane with the help of the adapter protein AP-2, it interacts with another adaptor protein, AP-3, that targets CD1d to the lysosomal compartments for lipid loading, unlike CD1a and CD1c (42, 44). This process is also dependent on the BH3 domain of BCL-XL, loss of which results in accumulation of CD1d in Rab7+ late endosomal compartments and failure to progress into acidic LAMP1+ lysosomal compartments (45).

Lipid loading onto to CD1d is a complex process that is not fully understood. It is generally accepted that additional lipid transfer proteins (LTPs), such as saposins A–D, Niemann-Pick C2 protein, and ganglioside monosialic acid 2 activator protein (GM2AP), are required to facilitate lipid exchange (46, 47). In addition to LTPs, CD1d lipid exchange is also thought to be highly dependent on the acidic pH of the lysosomal compartment. Indeed, computational modeling involving molecular dynamics showed that the CD1d antigen binding site becomes highly dynamic at pH 4.5, representative of the acidic conditions in late endosomes/lysosomes, which may facilitate lipid exchange. While not conclusive, the pair of computational studies provided some structural evidence for the formation of LTP/antigen/CD1d complexes at acidic pH. In addition, the studies suggest GM2AP can stabilize the binding site of apo-CD1d (CD1d without a lipid present), which normally has a collapsed binding site. In combination with the increased plasticity of the lipid binding at acidic pH, these data suggest a model in which LTPs are able to complex with and ultimately outcompete CD1d for binding its lipid antigen (47, 48). This could allow the currently loaded lipid to be removed with the CD1d binding site remaining stabilized and dynamic, thus providing the best conditions for a new lipid to be loaded before returning to the PM.

Type 1 iNKT cells make up the most important NKT cell subset for promoting anti-tumor immunity. Disruption of the recycling process through truncation of the tyrosine based signaling motif rendered APCs unable to activate type 1 NKT cell hybridomas *in vitro*, although they were still able to activate anti-inflammatory type II NKT cell hybridomas *in vitro* (49). Additionally, there is evidence that the altered metabolism of tumor cells results in the production of tumor antigens and enhanced CD1d antigen presentation. Activation of the AMPK pathway and induction of hypoxia inducible factors (HIF), common in malignancy and infection, using 2-deoxyglucose, cobalt chloride, AICAR and metformin result in enhanced CD1d mediated primary NKT cell activation *in vitro* (50). Similarly, induction of the pro-survival factor BCL-xL, common in early tumorigenesis, leads to enhanced CD1d mediated antigen presentation to NKT cells *in vitro* (45). Altogether, these studies support the idea that tumorigenesis results in danger signals, in the form of tumor-associated lipid antigens (the nature of which was not characterized in these studies), that are loaded onto CD1d during recycling and subsequently detected by NKT cells during cancer immune surveillance.

## The CD1d and lipid antigen interaction

Functional CD1d is composed of two components: the heavy chain and β_2_-microglobulin, similar to classic MHC I molecules. The heavy chain of CD1d is composed of three domains: α1, α2, and α3 (51). The α3 domain contains the transmembrane region and is anchored to the cell membrane. The α1 and α2 domains combine to form the antigen binding groove. Unlike the relatively wide peptide binding groove of MHC I, CD1d has a much narrower groove. However, the structure of CD1d contains two deep pockets below the entrance to the binding groove, which are called the A’ and F’ pockets and roughly correspond to their shallower counterparts in MHC I molecules (51). These deep pockets have unique shapes, with the A’ pocket having a long and curved shape compared to the more short and straight F’ pocket, and are lined with hydrophobic residues that can stabilize interactions with the hydrophobic acyl chains of lipid antigens. This structure results in the generally accepted configuration of the CD1d/lipid complex, with the hydrophobic acyl chains buried deep in the A’ and F’ pockets and the often hydrophilic head group exposed through the narrow binding groove for recognition by the TCR.

It is well known that the head groups of lipid antigens play a role in interacting with the NKT TCR (52, 53). East et al. reviewed the structural features of lipid antigen head groups that are not only essential for NKT cell activation, such as an α-linked sugar moiety, but can bias NKT cells towards either Th1 or Th2 profiles, referred to as structure-activity relationships (54). Interestingly, the acyl chain structure can also influence a lipid antigen’s ability to stimulate NKT cells, despite not interacting directly with the NKT-TCR. A series of experiments utilizing phosphatidylethanolamine (PE), a known CD1d-dependent lipid antigen that activates the 24.8.A NKT cell hybridoma, showed that increasing the degree of unsaturation up to 3 double bonds increased both the stability of binding as well as degree of NKT cell activation (52). Specifically, the double bonds had to be in the *cis* configuration. One possibility is that the *cis* double bond allows the acyl chain to effectively wrap around the curve in the A’ pocket. However, another study instead suggested that modulating the length and saturation of the acyl chain in the F’ pocket, not the A’ pocket, impacted binding affinity and NKT cell activation, with optimal characteristics being a chain length of 16-18 carbons and 1-2 unsaturations (55).

**Figure 3:**
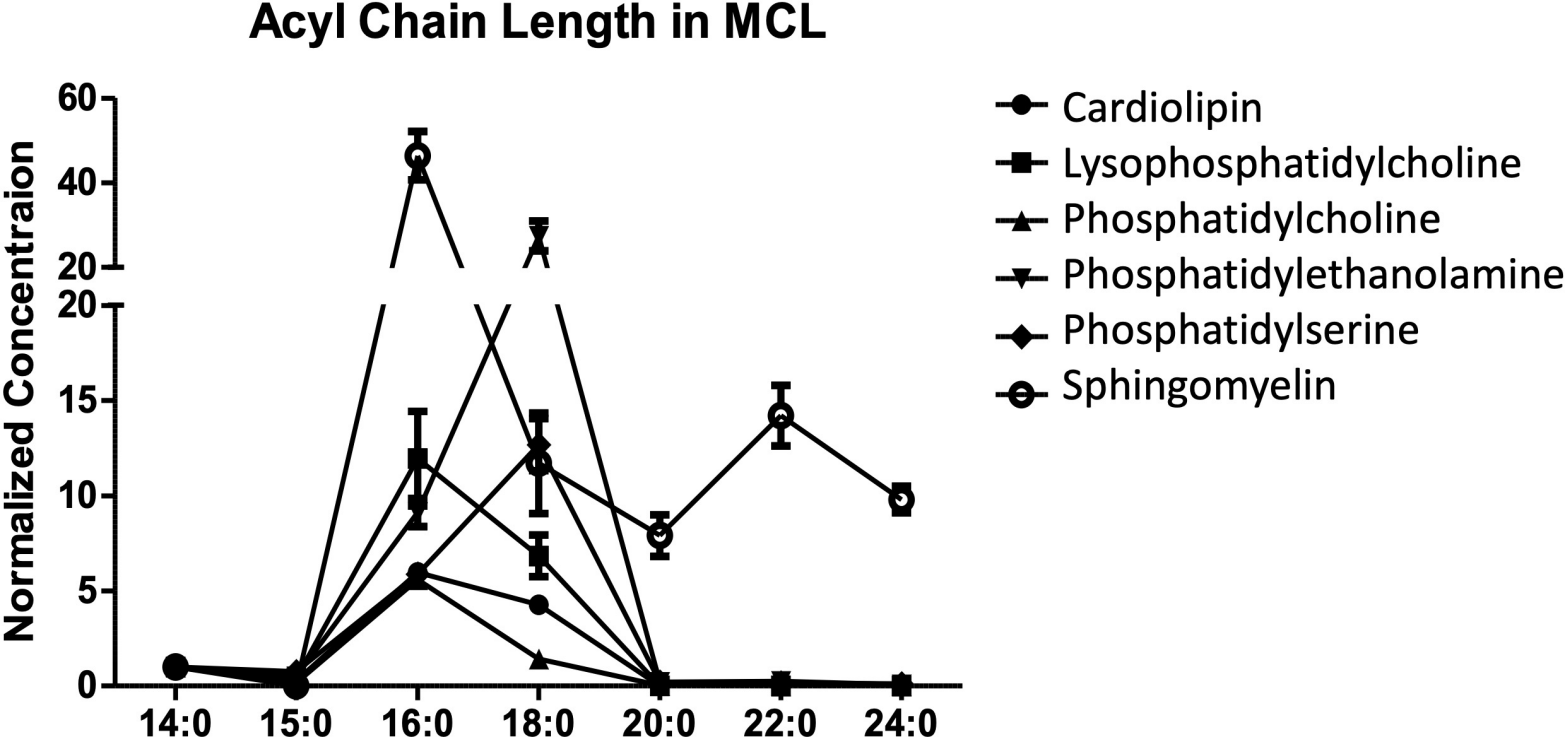
Acyl chain length in B cell lymphoma. The lipid profile of the human mantle cell lymphoma SP53 was assessed. The normalized concentration of different acyl chain lengths was determined for six different lipid classes. Three biological replicates were analyzed using differential mobility spectroscopy to determine the total number of moles of each lipid class present per billion cells. The average number of moles per billion cells for each of the indicated lipid species was assessed for acyl chain lengths. In our published studies, we focused on cardiolipin and are showing the other lipid data for comparison.

In our recent publication, we performed a lipidomic analysis of the human mantle cell lymphoma (MCL) line SP53 (56). Further examination of these data revealed that the majority of lipid species possessed 16 or 18 carbon long acyl chains, with 24 carbons being the longest acyl chain detected (**Figure 3**). The A’ pocket can accommodate acyl chains of up to 26 carbons while the F’ pocket can only accommodate up to 18 carbons. However, the A’ pocket is known to utilize 16 carbon long spacer lipids that can fill unfilled portions of the pocket while the F’ pocket does not (57). This could explain the relative importance of chain length in the F’ pocket compared to the A’ pocket, to neither over- or under-fill both pockets for optimal binding.

Why then would chain length and unsaturation result in more efficient NKT cell activation? One possibility is that optimal chain length and saturation not only influence CD1d binding affinity but also alter the structure of the TCR binding site. This is supported by evidence that only a partial filling of the F’ pocket by a 9-carbon acyl chain resulted in collapse of the unfilled portion that led to a ~2Å shift between residues Leu139 and Thr154 of the α2 helical domain main chain that makes up an essential portion of the binding groove. Given the precise nature of NKT TCR recognition, with the TCR CDR3 sitting parallel above the F’ pocket, any shift in the binding groove orientation could drastically alter NKT TCR binding affinity (55). Another possibility, which is not necessarily mutually exclusive with the other, is that unsaturation increases the efficiency of lipid loading. Double bonds in the *cis* conformation are much more flexible than their *trans* counterparts, which could help the acyl chain bind into the CD1d pockets more effectively than if they were rigid (52).

A third possibility is that acyl chain length has an impact on the type of response, given the ability of NKT cells to produce a variety of Th1, Th2, and Th17 cytokines, as opposed to whether there is any functional response. Indeed, a study on NKT cells from both mice and humans showed that acyl chain length impacted the ratio of IL-4 to IFN-γ produced, with shorter acyl chains leading to production of more IL-4 (a Th2 cytokine) than IFN-γ (a Th1 cytokine), possibly due to IFN-γ production requiring a longer duration of TCR binding facilitated by the more stable CD1d/lipid complex when longer acyl chains are present (58).

## The cancer lipidome

Keeping in mind the impact of lipid antigen structure on NKT cell responses, we will consider the lipid antigens that may be involved in NKT cell immunosurveillance of tumors. First, it is worth considering why we would expect tumors to be able to be identified based on lipid antigens. The idea that tumor cells have a unique lipidome when compared to healthy cells has long been accepted (59). Tumor cells have vastly different energy and signaling needs compared to healthy cells, and therefore would be expected to utilize different types of lipids to accomplish those needs. Indeed, enhanced *de novo* fatty acid biosynthesis, referred to as a lipogenic phenotype, is known to play a role in tumor development, with some going as far to say that it represents a hallmark of tumors (59, 60). *De novo* fatty acid biosynthesis is intricately linked to tumor progression through one of the most well-known cancer phenotypes, the Warburg effect.

Described by the Nobel Prize winning scientist Otto Warburg in 1953, the Warburg effect is the tendency of tumor cells to utilize lactic acid fermentation as opposed to oxidative phosphorylation even under aerobic conditions (61). The utility of the Warburg effect for the unique requirements of tumor cells is multi-faceted, but can be summed up by the idea that even more important to tumor cells than energy production, of which lactic acid fermentation is less efficient, is the need to make abundant material to support rapid growth. This is particularly true for fatty acids, which not only make up a significant portion of cell membranes in the form of phospholipids, but also lipid signaling molecules, acylated proteins, etc. By obtaining the majority of its energy through glycolysis, tumors can utilize citrate from the citric acid cycle to create acetyl-CoA, the main precursor for fatty acid synthesis, to generate fatty acids instead of completing the citric acid cycle (59). These fatty acids can then go on to help build the array of lipids necessary to support a growing tumor.

The notion that fatty acid biosynthesis is directly involved in promoting cancer pathogenesis aligns with observations that elevated levels of fatty acid synthase (FAS), the enzyme responsible for initial generation of fatty acid, correlates with poor outcomes in breast cancer patients (62). In contrast to normal cells, where the majority of free fatty acids are obtained from the diet and FAS activity is of little importance, tumor cells obtain the majority of their free fatty acids through FAS activity (60, 63). This has led to studies targeting FAS for inhibition in tumor models, such as in ovarian cancer, where inhibition of FAS resulted in activation of the AMPK pathway and subsequent cytotoxicity in the SKOV3 human ovarian cancer cell line (64).

Further evidence supporting the notion of a lipogenic phenotype correlating with tumor development came in a 2010 study examining the role of monoacylglycerol lipase (MAGL) in tumor pathogenesis (65). MAGL hydrolyzes monoacylglycerols in fat stores to release fatty acids for use in other cellular processes. MAGL overexpression resulted in a lipid signature consisting of elevated free fatty acids and subsequent complex fatty acids such as lysophospholipids and phosphatidic acid. Importantly, a loss of cancer aggressiveness that occurs with MAGL inhibition can be rescued by the addition of exogenous fatty acids, suggesting that these lipids are playing an essential role in promoting tumor pathogenesis. In agreement, MAGL levels were later shown to correlate with tumor formation in human hepatocellular carcinoma (HCC) patients (66). Additionally, these data provide mechanistic support for notion that high fat diets and obesity are linked to cancer risk.

More details on the composition of the cancer lipidome came in 2017 utilizing a mouse model where overactive mTORC2 signaling resulted in progressive liver disease culminating in HCC (67). In agreement with the previous study, tumor development was dependent on *de novo* fatty acid synthesis. In addition, the authors found the levels of more complex lipid species, including both glycophospholipids and sphingolipids, were elevated in mice progressing towards HCC. CD1d is able to present both glycophospholipids and sphingolipids. Together, these data leave open the possibility that NKT cells participate in cancer immune surveillance by recognizing and responding to the tumor lipidome.

**Figure 4:**
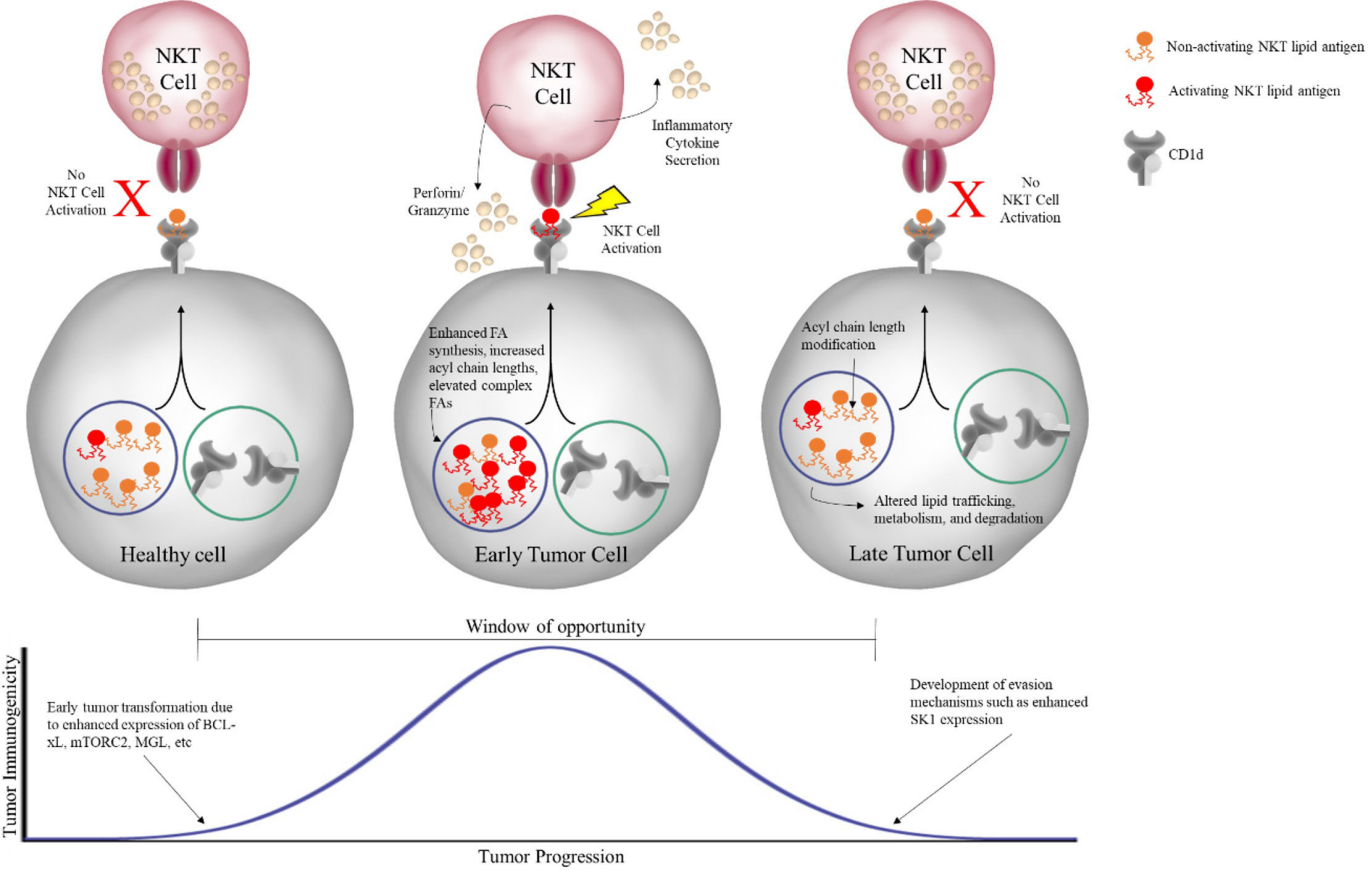
Window of opportunity for NKT cell-based immunotherapy. As healthy cells undergo early transformation into tumor cells, metabolic changes result in changes in the lipid profile of the cell. In the early stage, these tumor-associated lipids can be presented by CD1d on the cell surface to NKT cells, which subsequently respond both directly, through secretion of effectors like perforin/granzyme, and indirectly, through secretion of inflammatory cytokines, in order to eliminate the tumor. However, if the tumor is unable to be completely eliminated, reaching the equilibrium phase, it will acquire further changes that allow for evasion of NKT cell recognition, such as modification of acyl chain length and altered lipid processing, ultimately preventing activating lipid antigens from being presented to NKT cells.

If NKT cells can respond to tumor-associated lipid antigens, then we would also expect fully developed tumors to develop escape mechanisms to evade being targeted by NKT cells. This could involve several different processes, such as degrading the lipids before they can be presented or modifying their acyl chains so that they cannot be presented effectively. In fact, the Webb lab has long held the view that there is a window of opportunity early in tumor development where tumors are most susceptible to NKT cell-based immunotherapy. As illustrated in **Figure 4**, this window begins when healthy cells begin to undergo transformation into tumor cells, perhaps by enhanced expression of mTORC2 or MAGL as discussed earlier, but still can present tumor-associated lipids. In agreement, Subrahmanyam et al. showed that enhanced expression of BCL-xL, an anti-apoptotic member of the BCL-2 family of proteins often upregulated in early tumor development, results in increased NKT cell responses in a B cell lymphoma cell line (45). While the BCL-xL study did not focus on changes in the lipidome with enhanced BCL-xL expression, they did find that knockdown of BCL-xL resulted in reduced CD1d recycling and NKT cell responses. A reasonable conclusion would be that reduction in CD1d recycling leads to reduced NKT activation because tumor-associated lipids are unable to be loaded for presentation to NKT cells.

However, if the tumor continues to grow, we hypothesize that the window ultimately closes with the tumor acquiring the ability to hinder activating tumor-associated lipid antigens from NKT cell surveillance (**Figure 4**). As discussed in detail in our recent publication, the enzyme sphingosine kinase 1 (SK1), which is overexpressed in many different tumors, may participate in tumor cell evasion of NKT cells responses. Knockdown of SK1 in human MCL cell lines resulted in enhanced NKT cell responses, suggesting overexpression of SK1 is inhibiting NKT cells responses (56). Further, knockdown of SK1 resulted in a lipid profile characterized by a general increase in levels of several different classes of complex lipid species, similar to that seen in the previous mTORC2 and MGL studies. Interestingly, the study discussed previously utilizing overactive mTORC2 to facilitate HCC development in mice was able measure the expression of several lipids and enzymes, including SK1, over time as the mice progressed from healthy to HCC. They found that SK1 was unchanged early but upregulated late in disease progression, coinciding with the emergence of full-blown HCC (67).

Our knockdown studies revealed evidence for the role of acyl chain length in tumor recognition by NKT cells. Knockdown of SK1 resulted in a trend towards longer acyl chain lengths through an unclear mechanism, including a predominance of 16 and 18 carbon long acyl chains, the optimal length for CD1d loading (55, 56). In fact, increased acyl chain length appears to be a common and potentially targetable trait in lung squamous cell carcinoma (68). In addition, both hormone receptor-positive and triple-negative breast cancer show overexpression of some of the enzymes responsible for fatty acid chain elongation (69). When comparing the two types of breast cancer, it was found that triple-negative, the more aggressive of the two, actually had lower levels of 16:1 and 18: acyl chains. Given that these are the optimal chain lengths for CD1d loading, this leaves open the possibility that a portion of triple-negative breast cancer’s aggressiveness is due to better evasion of NKT cell responses, something that may be caused by overexpression of enzymes like SK1 late in tumor development.

We would not expect the majority of tumors in humans to be identified until after the window has begun to close, unless detected early by preventative screening, since they would likely not cause significant symptoms beforehand. However, detailing the interplay between NKT cells and tumors prior to the escape phase will continue to shed light on the body’s own natural protection mechanisms, allowing us to develop strategies to support and enhance existing systems. Studies that allow us to monitor the entirety of tumor development, such as in the mTORC2 HCC mouse study, provide vital information for future research.

Now that we have considered the cancer lipidome and general implications for NKT cell-based immunotherapy, we will next discuss individual lipid species that may serve as lipid antigens for NKT cell in the cancer lipidome.

## Lipid antigens for NKT cells in cancer

While there is strong evidence that tumor cells generate lipid species that act as activating antigens for NKT cells, serving as part of the foundation for how NKT cells participate in cancer immune surveillance, the nature of these species has not been fully elucidated. One possible reason is that lipid species often require elaborate maintenance and measurement techniques, such as a wide array of solvents (DMSO, chloroform:methanol mixtures, etc.) for maintenance and mass spectrometry for detailed measurement. Another possible reason is that most studies have been conducted in mice. However, mouse NKT cells recognize a more limited repertoire of antigens on human CD1d compared to human NKT cells (70), leaving open the possibility that some activating lipids for human NKT cells are not detected depending upon the experimental setup.

Nonetheless, identifying these antigens will have far-reaching implications, informing multiple different avenues of research. These include new information on both NKT cell and tumor cell biology and potential new therapies. With respect to NKT cell biology, examining the responses of NKT cells to tumor-derived lipids can potentially illuminate more details on the best type of response against tumors. With respect to tumor cell biology, working “backwards” to find how these antigens are produced may inform how cell metabolism is altered by tumorigenesis and novel mechanisms utilized by tumors to evade anti-tumor immunity. Finally, combining these insights will result in novel approaches to cancer immunotherapy as we both interfere with evasion mechanisms and drive NKT cell activation towards an optimal anti-tumor response.

A 2009 study sought to thoroughly characterize endogenous CD1d ligands (71). They generated a secreted form of CD1d by truncating the cytoplasmic and transmembrane regions. They then expressed the secreted form of CD1d in the human HLA class I deficient lymphoblast cell line 721.221. After collecting the secreted CD1d, lipids were extracted using a chloroform:methanol:water extraction and then characterized using high-performance liquid chromatography followed by tandem mass spectrometry (HPLC MS/MS). The identified lipids fell into two broad categories: phospholipids and sphingolipids. Among the phospholipids, mono-, di-, and tetra-acylated lipids were identified such as cardiolipin (CL), phosphatidylcholine (PC), phosphatidylethanolamine (PE), and phosphatidylserine (PS). Among the sphingolipids, both sphingomyelin and glycosylated sphingolipids (GSLs) were identified as CD1d ligands.

CL is a tetra-acylated phospholipid that is found in either the cell walls of gram-negative bacteria or in the inner-mitochondrial membrane of eukaryotic cells. In the mitochondria, cardiolipin stabilizes and helps “lubricate” ATP synthase, the enzyme that directly produces ATP from ADP (72). CL is known to bind to CD1d and stimulate CD1d-restricted γδ T cells in mice and possibly type 2 NKT cell line *in vitro* (73, 74). While a follow-up study from the same group that identified CD1d ligands did not find that cardiolipin could stimulate type 1 NKT cells, they utilized a CD1d-Fc fusion system to present to NKT cells, which may not effectively represent antigen processing in, and antigen presentation on, a live cell (75). In addition, acyl chain structure has been shown to change the ability of phospholipids to bind to CD1d (52). In fact, our recent publication found that cardiolipin was able to stimulate type 1 NKT cell activation; however, the ability of cardiolipin to activate NKT cells was highly dependent on the acyl chain structure, with 16:1 cardiolipin, but not 18:1 cardiolipin, able to activate (56).

It is worth noting that knockdown of SK1 in the MCL cell line SP53 resulted in increases in several different 16 and 18 carbon long CL species (56). This aligns with the HCC study where CL was found to be elevated in diseased mice (67). In that study, CL expression was driven by mTORC2 expression, as a separate model driven by mTORC1 overexpression did not result in increased CL expression and was not as aggressive in terms of tumor development, and it was postulated that CL helped improve mitochondrial function to support tumor cell growth. A last point that was that CL was overexpressed early in tumor development, and levels decreased prior to HCC formation. While not noted in the original study, this is particularly interesting as it aligns with our conclusion that the SK1-mediated decrease in CL helped to close the window of opportunity for NKT cell recognition by eliminating a potential activating lipid from being presented to NKT cells.

While recent evidence supports a role of cardiolipin in NKT cell responses against cancer, there remains the intriguing question of how a tetra-acylated lipid species is able to bind to CD1d. The majority of lipids we have considered are di-acylated or, in the case of lyso-derivatives, mono-acylated. In both of those situations, all of the acyl chains are able to bind a pocket of CD1d, protecting the hydrophobic acyl chains and leaving the polar head group exposed to the solvent. However, given that cardiolipin has four acyl chains, it is worth asking what happens to the other two acyl chains during CD1d binding. One possibility is that cardiolipin binds to two molecules of CD1d present on the surface of the same cell, which would add an intriguing layer of complexity and suggest a role for lipid rafts. However, a 2011 study that identified the cardiolipin-CD1d complex as a ligand for γδ T cells was also able to generate a crystal structure of cardiolipin bound to CD1d (73). While they were unable to construct an electron density for two of the four acyl chains, they found that there were only two acyl chains bound to a single CD1d molecule and also identified gaps in the crystal lattice in the solvent region in which two additional acyl chains could fit. Altogether, this suggested that cardiolipin binds to a single CD1d molecule with two of the acyl chains essentially just hanging out in the solvent. The implications of this structure, and whether or not it holds true *in vivo*, are not entirely clear.

Another interesting question is how SK1 impacts cardiolipin levels. SK1 catalyzes the addition of a phosphate group to sphingosine in order to generate S1P. This leaves two distinct possibilities for how it would ultimately impact cardiolipin levels. The first is relatively straightforward. Despite the fact that there are elevated levels of free fatty acids in tumor cells, the pool is still limited. Thus, increased production and secretion of S1P could deplete the amount of fatty acids available to generate other lipid species. This agrees with our data from SK1 knockdown in B cell lymphoma, in which we observed a general increase in all lipid species after SK1 knockdown (56).

Another possibility revolves around S1P signaling and mitochondrial function, which may provide insight to the related question of how cardiolipin gains access to CD1d given its location in the inner mitochondrial membrane. Knockdown of SK2, which also catalyzes the production of S1P, has been shown to have a detrimental impact on mitochondrial function, an effect that was mediated through the S1P receptor 1 (76). In addition, cardiolipin is known to localize from the inner mitochondrial membrane to the outer mitochondrial membrane in times of mitochondrial stress, where it may facilitate the induction of mitophagy (77). Mitophagy is the controlled degradation of mitochondria and, while it has not been shown, it is possible that it may expose cardiolipin to lysosomal compartments where it could be loaded on CD1d for presentation. Thus, knockdown of SK1 and corresponding reduction in S1P signaling could result in mitochondrial dysfunction and increased rates of mitophagy leading to an increase in cardiolipin presentation.

A third situation, not mutually exclusive with the other two, combines both reaction kinetics and mitochondrial dysfunction. Specifically, ceramide is a precursor to sphingosine. Therefore, enhanced SK1 expression should drive the production of S1P and ultimately reduce the levels of ceramide as sphingosine is depleted. In addition, ceramide is known to induce lethal levels of mitophagy through direct binding to LC3B-II, which recruits the autophagosome to the mitochondria thereby initiating mitophagy and ultimately cell death (78). Thus, enhanced SK1 expression may have a multi-faceted approach that depletes potentially activating lipids, inhibits mitophagy, and protects mitochondrial function to promote cell growth. Taken together, these data further suggest studies focused on examining CD1d-mediated antigen presentation from an ecological evolutionary developmental biology (eco-evo-devo) perspective.

PC is a di-acylated phospholipid with choline as a head group found in the outer leaflet of cell membranes. PC stimulates IFN-γ production in a CD1c-dependent manner to intestinal γδ T cells (79). In addition, the PC derivative lysophosphatidylcholine (LPC), is part of the lipid network that is upregulated by MAGL, which promotes a tumorigenic lipid profile (65). We also found that 16 and 18 carbon forms of PC were upregulated in a B cell lymphoma cell line with knockdown of SK1 (data not shown) (56). The fact that LPC is elevated in early tumors identifies it a promising candidate as an activating antigen for NKT cells. However, a study in multiple myeloma found that while LPC was indeed present in human patients, it preferentially stimulated type II NKT cells with a tendency towards IL-13 secretion (80–82). This is more suggestive of LPC being utilized by cancer to regulate immune activation in the face of tumor induced inflammation. In agreement, mTORC2, which is known to promote tumorigenesis, was found to promote the synthesis of LPC in a mouse model of HCC (67). Interestingly, we found that SK1 knockdown did not result in an increase in LPC, unlike PC, but may actually result in a decrease in 16 and 18 carbon forms of LPC (which we did not show in our paper) (56). If LPC does indeed lead to an anti-inflammatory phenotype, SK1 activity could promote tumor evasion by actually increasing the intra-cellular levels of LPC. Therefore, while not likely to be a lipid antigen utilized in anti-tumor therapy, it may be possible to inhibit LPC’s immune regulatory function to release the breaks on anti-tumor therapy.

PE is a di-acylated phospholipid with an ethanolamine head group found in cell membranes where they may play a role in membrane fusion. Similar to PC, both of which are antigens found in pollen, the effects of PE seem to be mixed. PE is able to activate murine NKT cell hybridomas, where increased unsaturation of acyl chains facilitates activation (52, 83). In addition, PE is able to activate γδ T cells to produce both IFN-γ and IL-4 (84). However, in another study looking at activation of duodenal γδ T cells found that PE promoted a more regulatory cytokine profile dominated by IL-4 and IL-13 (79). Again, similar to PC, PE is promoted by mTORC2 and MGL in cancer cells (65, 67). PE also showed a relatively large increase in 16 and 18 carbon species upon knockdown of SK1 in a B cell lymphoma cell line (data not shown) (56). Thus, while PE appears to fit as a tumor-associated lipid that could serve as an antigen for NKT cells, the mixed responses it elicits from NKT cells make it difficult to utilize.

PS is a di-acylated phospholipid with a serine head group found in the cell membrane where it plays a role in cell cycle regulation and apoptosis. While it has been found to be a ligand for CD1d, is also included within the lipid network promoted by mTORC2, and is slightly elevated by SK1 knockdown (data not shown), there is not much evidence for or against its role as an NKT cell antigen in cancer (56, 67). Interestingly, NKT cells are known to express Tim-1 and Tim-3, surface receptors that recognize PS on the surface of apoptotic cells (85). Tim-1 and Tim-3 may provide opposing stimulatory and inhibitory signals, respectively, providing a TCR independent mechanism for NKT cells to respond to apoptotic cells.

Sphingolipids are a diverse class of lipids that are of great interest due to their multi-faceted role in the immune system. In addition to their ability to bind to CD1d and potentially act as an antigen for NKT cells, they have been shown to directly play a role in early development and establishment of NKT cell populations in the colon (86). Sphingolipids are built upon a sphingoid base backbone. The common natural sphingoid bases are sphingosines, sphinganines, and phytosphingosines (87). The ceramides are the next level of sphingolipids in terms of complexity. Ceramides are *N-*acylated sphingoid bases that have a simple hydrogen for a head group. The final level, in terms of complexity, are the complex sphingolipids, which have a more complex head group attached to ceramide. Two main categories of complex sphingolipids that were found to bind to CD1d include sphingomyelin and glycosphingolipids (GSLs) (71).

Sphingomyelin is a complex di-acylated sphingolipid composed of ceramide with either a phosphocholine or phosphoethanolamine head group. Sphingomyelin is involved in multiple different biological processes, often altering cell signaling through its involvement in lipid rafts. Sphingomyelin has also been found to be dysregulated in cancer. mTORC2, which supports tumorigenesis, promotes *de novo* sphingolipid synthesis (67). In addition, sphingomyelin was found to be upregulated in mouse breast cancer cell lines compared to non-cancerous cells; however, this was not the case for human cells (88). On the other hand, another study looking at a human primary colon cancer cell line compared to its lymph node metastasis counterpart found that sphingomyelin was decreased (89). This may suggest that sphingomyelin levels change with progressing tumor development, starting off high and later decreasing, making it an interesting target for future study.

While sphingomyelin was identified as part of the pool of CD1d ligands, early evidence suggested that it was not able to stimulate NKT cells (71, 75). This is in agreement with a recent study that suggested a regulatory role for sphingomyelin in NKT cell activation. Specifically, knockout of ASM, the enzyme responsible for sphingomyelin degradation, led to reduced CD1d-mediated NKT cell activation resulting in loss of NKT cell selection in the thymus. These data suggest that sphingomyelin blocks the CD1d-loading of activating lipids thus preventing NKT cell activation (90). However, further analysis found that lyso-sphingomyelin, a mono-acylated derivative of sphingomyelin, was able to stimulate NKT cells, although very weakly (75). In B cell lymphoma, the expression of different species of sphingomyelin was limited compared to other lipids, complicating the conclusions about the effect(s) of SK1 knockdown (data not shown) (56). If sphingomyelin indeed plays a regulatory role for CD1d-mediated NKT cell activation, future studies should examine the expression of ASM in tumors with the expectation that downregulation of ASM would promote tumor immune evasion of NKT cell responses.

GSLs have various sugars as head groups on ceramide and include a variety of compounds such as cerebrosides (single glucose or galactose head group) and gangliosides (three or more sugars, one of which being sialic acid). The ability of a GSL to be an activating lipid for NKT cells is well known, given that α-GalCer, the canonical activating lipid for NKT cells, is a GSL (91). In addition, multiple GSLs including isoglobotrihexosylceramide (iGb3), gangliosides, and β-glucosylceramide (β-GlcCer) have been proposed to act as endogenous ligands for NKT cells, an area that is currently poorly understood (92–96). Recent evidence suggests that these antigens are not presented in their native forms, but require processing induced by PRR engagement on APCs (97).

With respect to cancer therapy, GSLs have shown potential utility. Chemical derivatives of iGb3 have been found to stimulate NKT cells in a CD1d-dependent manner and even skew the NKT cell response towards a more anti-tumor Th1 response dominated by IFN-γ secretion (98). In addition, the ganglioside NGcGM3 is not expressed in normal human tissues due to a genetic mutation in an enzyme involved in its synthesis. However, cancers can acquire changes that allow for the production of NGcGM3, making it a tumor-specific antigen. NGcGM3 stimulated NKT cell activation via CD1d presentation on B cells, making it an interesting target for CD1d+ B cell lymphomas (94).

While gangliosides such as iGb3 and NGcGM3 have promise as activating antigens for NKT cells, other gangliosides may actually inhibit NKT cell activation. Specifically, gangliotriaosylceramide (Gg3Cer), was found to be shed by the T cell lymphoma line L5178Y-R (99). While L5178Y-R cells did express CD1d on their surface, they were unable to stimulate NKT cells; however, inhibition of ganglioside shedding was able to restore NKT cell’s ability to respond to L5178Y-R. Exogenous addition of Gg3Cer to CD1d expressing mouse fibroblast was able to inhibit their ability to activate NKT cells, confirming the role of Gg3Cer as an inhibitory ganglioside for CD1d-mediated NKT cell activation. GD3 is a second ganglioside that has generated significant interest as it is expressed in various cancers. Early studies found that NKT cells produced high levels of IL-4 in addition to IFN-γ and IL-10 in mice vaccinated with either a GD3+ melanoma cell line or GD3-loaded APCs (95). Later studies in ovarian cancer found that GD3 is present in ovarian cancer ascites and can inhibit CD1d-mediated activation of NKT cell hybridomas (100). Together these studies suggest GD3 may mediate cancer immune evasion by failing to induce a strong Th1-type response.

## Conclusions and future directions

Ultimately, while a significant number of studies have been devoted to identifying NKT cell antigens in the context of anti-tumor immunity, there are many unknowns. Whereas some tumor associated NKT cell activating antigens have been identified (cardiolipin, PC, iGb3 and NGcGM3), a significant proportion of the antigens seem to be immune regulatory, i.e., tumors may be skewing malleable NKT cells to generate anti-inflammatory pro-tumorigenic responses (LPC, Gg3Cer, and GD3). Finally, other antigens identified operate through unique mechanisms such as sphingomyelin outcompeting other lipids for CD1d binding and PS highlighting the innate-like properties of NKT cells by signaling through PRRs on their surface. Despite the intricacies of these unique cells, this review demonstrates the significant strides made in our understanding of the mechanisms that can be utilized to drive their anti-tumor properties. Continued research into tumor-associated lipid antigens for NKT cells will allow for the development of therapies that constrain the negative influences on NKT cell-based anti-tumor immunity and fully unleash these cells in a way that tumors simply cannot avoid.

## Author contributions

ML designed and wrote the review. TW provided guidance, reviewed, and edited the manuscript. All authors contributed to the article and approved the submitted version.
